# Glutathione S-Transferase Gene Polymorphisms and Treatment Outcome in Cervical Cancer Patients under Concomitant Chemoradiation

**DOI:** 10.1371/journal.pone.0142501

**Published:** 2015-11-16

**Authors:** Mohammad Abbas, Vandana Singh Kushwaha, Kirti Srivastava, Monisha Banerjee

**Affiliations:** 1 Molecular and Human Genetics Laboratory, Department of Zoology, University of Lucknow, Lucknow -226007, Uttar Pradesh, India; 2 Department of Radiotherapy, King George’s Medical University, Lucknow-226003, Uttar Pradesh, India; Queen's University Belfast, UNITED KINGDOM

## Abstract

**Purpose:**

Cisplatin based concomitant chemoradiation (CRT) is the standard treatment for locally advanced cervical cancer (CC). Glutathione S-transferase (GST), a phase II antioxidant enzyme is induced by oxidative stress generated by drugs and reactive oxidants. The present study was undertaken to evaluate the association of *GSTM1*, *T1* and *P1* polymorphisms with the outcome of CRT treatment in CC patients.

**Methods:**

A total of 227 cervical cancer patients with stages IIB-IIIB treated with the same chemoradiotherapy regimen were enrolled and genotyped for *GSTM1*, *T1* and *P1* gene polymorphisms by multiplex polymerase chain reaction (mPCR) and PCR-restriction fragment length polymorphism (PCR-RFLP). Overall survival was evaluated using Kaplan-Meier survival function and Cox proportional hazards model. All data were analyzed using SPSS (version 21.0).

**Results:**

Stratified analysis showed that *GSTM1* null (M1-) genotype was associated with a significantly better survival among patients with stage IIB cervical cancer (log-rank *P* = 0.004) than cases with stage IIIA/IIIB. Death and recurrence were significantly higher in patients with *GSTM1* present genotype (M1+) (*P* = 0.037 and *P* = 0.003 respectively) and those with M1- showed reduced hazard of death with an adjusted hazard ratio ‘HR’ of 0.47 (95% CI, 0.269–0.802, *P* = 0.006). Women with M1- genotype as well as in combination with *GSTT1* null (T1-), *GSTP1* (AG+GG) and *GSTT1* null/*GSTP1* (AG+GG) showed better survival and also reduced risk of death (HR = 0.31, *P* = 0.016; HR = 0.45, *P* = 0.013; HR = 0.31, *P* = 0.02 respectively).

**Conclusions:**

To the best of our knowledge, this is the first study to correlate the association of *GSTM1*, *T1* and *P1* gene polymorphisms with treatment outcome of CRT treated CC patients. Our results suggested that individuals with *GSTM1* null genotype and in combination with *GSTT1* null and *GSTP1* (AG+GG) had a survival advantage. Such genetic studies may provide prognostic information in CRT treated CC patients.

## Introduction

Cisplatin based concomitant chemoradiation (CRT) is the standard treatment used for patients with locally advanced cervical cancer. It is known to improve survival and reduce the recurrence rate [1–2]. However, primary or acquired chemoradioresistance are serious clinical problems that contribute to disease recurrence, progression and mortality [3–4]. Local and distant relapses occur due to the survival of some cancer cells leading to treatment failure. Therefore, new and more effective approaches are required to tackle this issue. The mechanisms behind the heterogeneous responses to treatment among patients are multifactorial and involve variability in genetic constitution. There is mounting evidence that genes involved in metabolism, DNA repair and intracellular signaling pathway can influence individual variability to treatment response and toxicity [5–6]. There is a constant search for new prognostic and predictive factors in order to individualize treatment regimens.

The cytotoxic effects of both radiation and chemotherapy are mediated primarily through generation of reactive oxygen species (ROS) and their byproducts causing cell damage. The tumor cell death by ROS is either by direct cytotoxic effect or intracellular apoptotic pathways. Therefore, various enzymes affecting ROS levels are likely to impact patient prognosis after treatment [7–8]. Glutathione S-transferase (GST) is a phase II metabolic enzyme that plays an important role in cellular defense against numerous harmful chemicals produced both exogenously and endogenously [9–10]. It protects cells from oxidative damage by catalyzing conjugation of ROS [11–12] and also detoxifies various chemotherapeutic agents such as alkylating agents, platinum compounds and adriamycin [3]. Glutathione (GSH) is one of the main detoxifying agents which can be affected by metabolic enzymes that use tripeptides as substrates. At the time of chemotherapy, sufficient activity of GST is crucial, since during the enzyme catalyzed conjugation reaction with GSH, the water solubility of drugs and other toxic materials increases and can be eliminated from the body [13–14]. Many studies have shown that genetic variation in these enzymes, including *GSTP1* A313G (rs1695, Ile105Val) and whole gene deletions in *GSTM1* and *GSTT1* have impact on survival and toxicities in different cancers treated with platinum agents and radiation [9, 15–17].

Due to the free radical scavenging activity and detoxification of platinum agents, role of GST in treatment response and survival in cervical cancer patients is plausible. In the present study, we have investigated patients’ survival and disease recurrence in relation to *GSTM1*, *T1* and *P1* gene polymorphisms after treatment with cisplatin based concomitant chemoradiation (CRT).

## Materials and Methods

### Ethics Statement

Cervical cancer patients who were under cisplatin based concomitant chemoradiation (CRT) at the Radiotherapy Department of King George’s Medical University (KGMU), Lucknow, India (from 2009 through 2011) were included in this study. This study was approved by the Institutional Ethics Committee of KGMU and all patients provided written informed consent for their participation in the study.

### Patients Selection and Sample Collection

Five milliliters venous blood samples were obtained from 244 subjects at the start of treatment regimen. The patients were of same ethnicity, stages IIB-IIIB according to International Federation of Gynecology and Obstetrics (FIGO), with no associated co-morbid conditions and exposed to first course treatment of CRT. Demographic and clinical characteristics of patients were obtained from medical records while staging and clinical diagnosis of patients were performed by expert clinicians as per guidelines of FIGO.

### Treatment and Response Evaluation

Chemotherapy and radiation dose were same for all patients. They received pelvic external beam radiotherapy (for a total dose of 50 Gy in 25 fractions) with weekly concomitant cisplatin (40 mg/m^2^) followed by three applications of high dose rate (HDR) intracavitory brachytherapy 7 Gy/fraction at one week interval. The patients who did not complete the planned chemoradiation dose or violated the treatment protocol were excluded from the study. The response to treatment was measured by RECIST criteria version 1.0 after one month of treatment. Women were followed-up after treatment and checked for survival. The primary endpoint was overall survival (OS) from the date of diagnosis to the date of death from any cause. Women who were alive at the end of the study were censored.

### Genotyping

Genomic DNA was extracted from peripheral blood samples by standard salting out method with slight modifications [18–19], checked on 1% agarose gel and quantified in a biophotometer (Eppendorf, Germany).

*GSTM1* and *T1* null genotypes (M1- and T1-) were detected by using multiplex polymerase chain reaction (mPCR) using specific primers. The *GSTP1* 313A>G polymorphism was genotyped by PCR-Restriction Fragment Length Polymorphism (PCR-RFLP) using specific primers and *Alw26*I restriction enzyme [20].

### Statistical Analysis

Demographic and clinical information was compared across genotypes using χ^2^ analysis and Fisher’s exact test. Genotype effects on overall survival (OS) were evaluated by Kaplan-Meier function and Cox proportional hazards model. The differences in OS across different genotypes were compared using the log-rank test. Hazard ratios (HRs) and 95% Confidence Intervals (CIs) were estimated using multivariate Cox proportional hazards model/Cox regression analysis with adjustment for age, pathological stage and histopathology. All *P* values were two-sided and differences were considered statistically significant for *P*<0.05. The statistical analyses were performed by SPSS (Version 21.0).

## Results

Among 244 cervical cancer patients enrolled, 227 were included in the study. Seventeen patients had protocol violations, out of which three did not commence treatment, two did not receive concomitant chemotherapy, one received additional chemotherapy and eleven did not complete treatment.

### Clinical Characteristics of Patients

The mean age of patients was 49.0±8.68 years. Histopathological analysis showed 216 patients (95.2%) with squamous cell carcinoma and remaining 11 (4.8%) with adenocarcinoma. The distribution of squamous cell carcinoma into three categories were 96 well (44.4%), 79 moderate (36.6%) and 21 poor (9.7%) while no differentiation was reported in histopathological report of 20 cases (9.3%). The staging of tumor according to FIGO was 117 cases (51.5%) with stage IIB and 110 (48.5%) with stage IIIA+IIIB (Table 1).

**Table 1:** Demographic and clinicopathological characteristics of cervical cancer patients (n = 227), and their distributions by genotypes of *GSTM1*, *T1* and *P1*.

Patients, n (%)		*GSTM1*, n (%)			*GSTT1*, n (%)			*GSTP1*, n (%)		
		M1+	M1 -	*P* value	T1+	T1-	*P* value	AA	AG+GG	*P* value
**Total**	227	147 (64.8)	80 (35.2)		176 (77.5)	51 (22.5)		128 (56.4)	99 (43.6)	
**Age (years)**										
31–40	53 (23.3)	32 (14.1)	21 (9.2)		34 (15.0)	19 (8.4)		31 (13.7)	22 (9.7)	
41–50	105 (46.3)	69 (30.4)	36 (15.9)	0.596	89 (39.2)	16 (7.0)	0.288	61 (26.9)	44 (19.4)	0.304
51–60	50 (22.0)	34 (15.0)	16 (7.0)		39 (17.2)	11 (4.8)		28 (12.3)	22 (9.7)	
>60	19 (8.4)	12 (5.3)	7 (3.1)		14 (6.2)	5 (2.2)		8 (3.5)	11(4.8)	
**Stage**										
IIB	117 (51.5)	73 (32.1)	44 (19.4)		88 (38.8)	29 (12.7)		59 (26.0)	58 (25.5)	
IIIA+IIIB	110 (48.5)	74 (32.6)	36 (15.9)	0.442	88 (38.8)	22 (9.7)	0.389	69 (30.4)	41 (18.1)	0.063
**Histology**										
SCC	216 (95.2)	142 (62.6)	74 (32.6)		169 (74.4)	47 (20.7)		126 (55.5)	90 (39.6)	
AD	11 (4.8)	5 (2.2)	6 (2.6)	0.18	7 (3.1)	4 (1.8)	0.267	2 (0.9)	9 (4.0)	**0.02**
**Vital Status**										
Alive	156 (68.7)	94 (41.4)	62 (27.3)		117 (51.5)	39 (17.2)		83 (36.6)	73 (32.2)	
Deceased	71 (31.3)	53 (23.4)	18 (7.9)	**0.037**	59 (26.0)	12 (5.3)	0.178	45 (19.8)	26 (11.4)	0.153
**Recurrence**										
Disease Free	160 (70.5)	92 (62.6)	68 (85.0)		120 (68.2)	40 (78.4)		86 (67.2)	74 (74.7)	
Never Disease Free	25 (11.0)	22 (15.0)	3 (3.8)	**0.003**	24 (13.6)	1 (2.0)	0.817	17 (13.3)	8 (8.1)	0.347
Recurred	42 (18.5)	33 (22.4)	9 (11.3)		32 (18.1)	10 (19.6)		25 (19.5)	17 (17.2)	

Bold, significant association (*P*<0.05); (+), present; (^_^), null; SCC, Squamous cell carcinoma; AD, Adenocarcinoma.

### Genotyping

The distribution of *GSTM1* genotypes showed 64.8% cases with *GSTM1* present (M1+) and 35.2% with *GSTM1* null (M1-) while distribution of *GSTT1* showed 77.5% cases with *GSTT1* present (T1+) and 22.5% with *GSTT1* null (T1-). The distribution of *GSTP1* genotypes showed 56.4% cases with AA, 43.6% with AG+GG. Adenocarcinoma of cervix was significantly higher in the patients having AG+GG genotype of *GSTP1* (*P* = 0.02). However, distribution of *GSTM1*, *T1* and *P1* did not (*P*>0.05) show significant association with age and clinical stage (Table 1).

### Survival of patients

The median follow-up duration for all patients was 34 months (range, 4.2–63.0 months). During the study period (2009–2011) 31.3% patients succumbed to death. The median survival had not been reached and overall mean survival time was 49.4 months (95% CI = 46.78 to 52.01). It was observed by stage-stratified analysis that the overall survival among patients with stage IIB was significantly better than in those with stage IIIA/IIIB in case of *GSTM1* null (M1-) individuals (log-rank *P* = 0.004). Disease recurrence was recorded for 42 women (18.5%). Most of the patients with *GSTM1* null genotype (M1-) were alive and disease free at the end of study period (*P* = 0.037 and *P* = 0.003, respectively) while none with *GSTT1*&*P1* genotypes exhibited similar response (Table 1). The multiple testing for all three studied genes with clinicopathological characteristics showed that the risk of recurrence was significantly reduced in *GSTM1* null/*GSTT1* null and *GSTM1* null/*GSTP1* AG+GG (*P* = 0.004 and *P* = 0.021 respectively). None of the other combinations showed any significant association.

### Effect of GST genotypes on Survival

**Fig 1:**
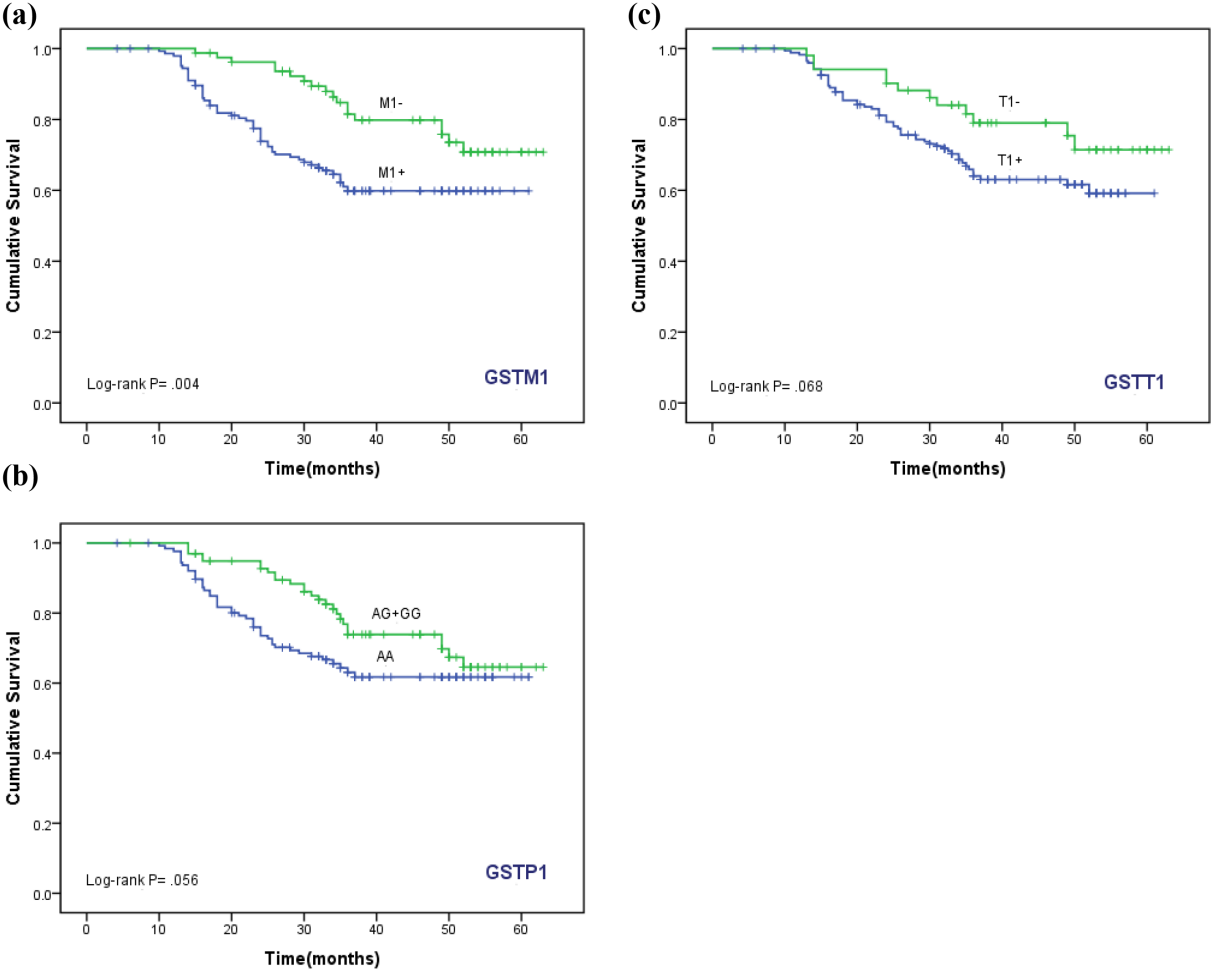
Kaplan-Meier function for overall survival among women treated for cervical cancer, by *GSTM1*, *T1* and *P1* genotypes. Survival difference by log-rank test.

**Fig 2:**
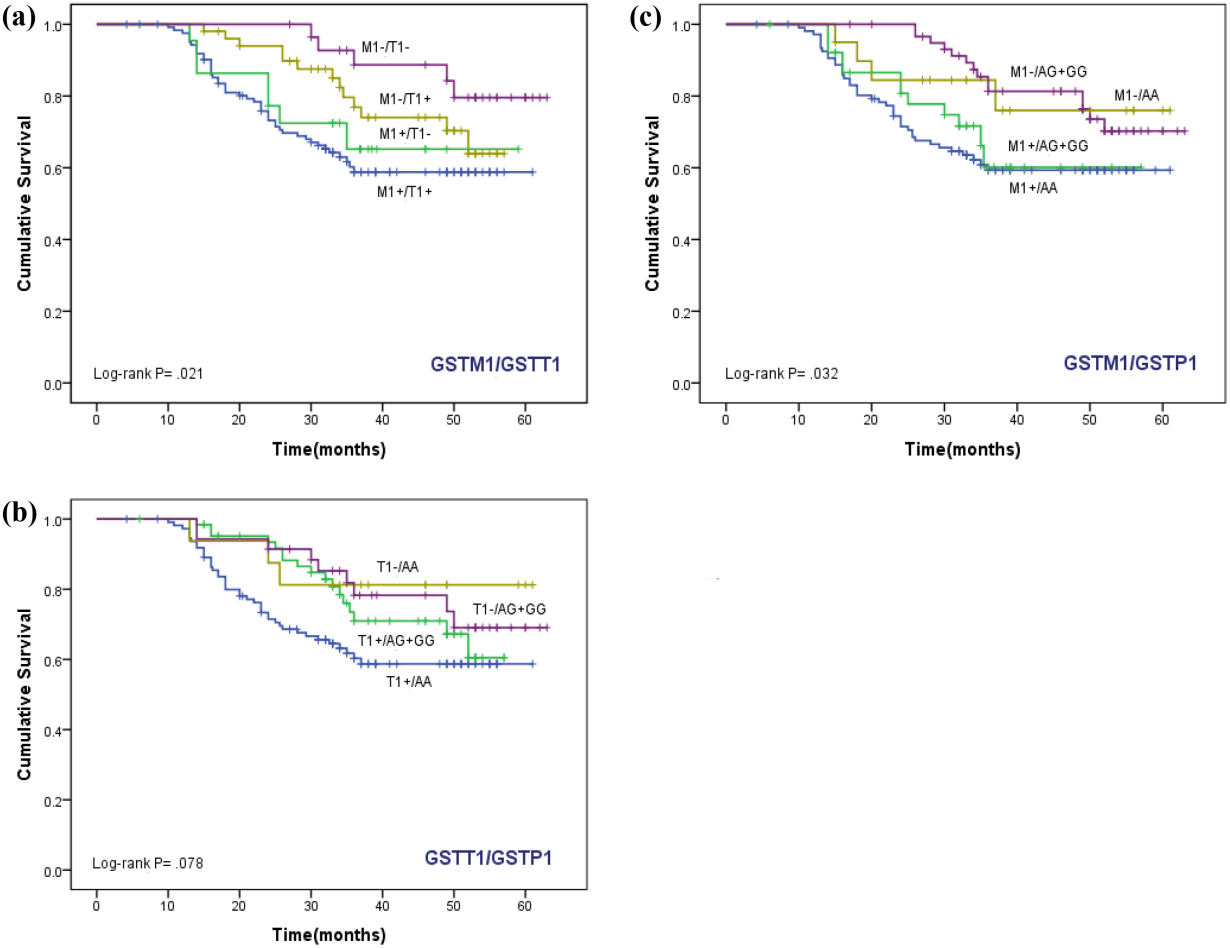
Kaplan-Meier function for overall survival among women treated for cervical cancer, by *GSTM1*, *T1* and *P1* genotypes in combination. Survival difference by log-rank test.

The Kaplain-Meier function and Log rank test for survival in cases with *GSTM1*, *T1* and *P1* genotypes are shown in Figs 1 and 2. In the Kaplain-Meier curve, *GSTM1* null genotype (M1-) was associated with better overall survival (log-rank, *P* = 0.004). The overall survival in women with *GSTM1* null (M1-) genotype and *GSTT1* null (M1-/T1-) and *GSTP1* (M1-/AG+GG) was significantly better (log-rank, *P* = 0.021 and *P* = 0.032 respectively).

Association of *GSTM1*, *T1* and *P1* genotypes with overall survival as analysed by Cox proportional hazards model, adjusted for age, stage and histopathology is shown in Table 2. There was significant reduction in hazard of death to 0.47 among women with *GSTM1* null genotype (M1-) when compared with women having *GSTM1* present (M1+) genotype (95% CI, 0.269–0.803, *P* = 0.006). There was no significant association with *GSTT1* null and *GSTP1* AG+GG genotypes. In addition, the combined effect showed that all three genes have impact on survival. Women with *GSTM1* null genotype in combination with *GSTT1* null (M1-/T1-) and *GSTP1* (M1-/AG+GG) showed further reduced risk of death [hazard ratio adjusted (HR) = 0.31, 95% CI, 0.121–0.805, *P* = 0.016; 0.45, 95% CI, 0.243–0.846, *P* = 0.013 respectively]. The adjusted HR associated with triple GST genotype combinations have been shown in Table 2. The analysis showed better survival in patients having *GSTM1* null/*GSTT1* null/*GSTP1* AG+GG genotype combination (HR = 0.31, 95% CI, 0.120–0.836, *P* = 0.02).

**Table 2:** Associations between *GSTM1*, *T1* and *P1* genetic polymorphisms and survival after treatment (CRT) for cervical cancer.

Genotypes	No. of cases, n (%)	Deaths, n (%)	HR^a^ (95% CI)	*P* value
**Total**	227	71		
***GSTM1***				
M1+	147 (64.8)	53 (74.6)	1.0 (Ref)	
M1-	80 (35.2)	18 (25.4)	0.465(0.269–0.802)	**0.006**
***GSTT1***				
T1+	176 (77.5)	59 (83.1)	1.0 (Ref.)	
T1-	51 (22.5)	12 (16.9)	0.598(0.318–1.123)	0.11
***GSTP1***				
AA	128 (56.4)	45 (63.4)	1.0 (Ref.)	
AG +GG	99 (43.6)	26 (36.6)	0.639(0.386–1.060)	0.083
***GSTM1/GSTT1***				
M1+/T1+	125(55.0)	46 (64.8)	1.0 (Ref.)	
M1+/T1-	22(9.7)	7 (9.9)	0.828(0.373–1.839)	0.643
M1-/T1+	51(22.5)	13 (18.3)	0.539(0.291–0.999)	0.05
M1-/T1-	29(12.8)	5 (7.0)	0.312(0.121–0.805)	**0.016**
***GSTM1/GSTP1***				
M1+/AA	108 (47.6)	41 (57.8)	1.0 (Ref.)	
M1+/AG+GG	39 (17.2)	12 (16.9)	0.851(0.429–1.689)	0.645
M1-/AA	20 (8.8)	4 (5.6)	0.434(0.155–1.213)	0.111
M1-/AG+GG	60 (26.4)	14 (19.7)	0.453(0.243–0.846)	**0.013**
***GSTT1/GSTP1***				
T1+/AA	112 (49.3)	42 (59.2)	1.0 (Ref.)	
T1+/AG+GG	64 (28.2)	17(23.9)	0.633(0.352–1.139)	0.127
T1-/AA	16 (7.1)	3 (4.2)	0.461(0.141–1.508)	0.201
T1-/AG+GG	35 (15.4)	9 (12.7)	0.526(0.251–1.100)	0.088
***GSTM1/GSTT1/GSTP1***				
M1+/T1+/AA	95 (41.9)	38 (53.5)	1.0 (Ref.)	
M1+/T1+/AG+GG	30 (13.2)	8 (11.3)	0.710 (0.315–1.599)	0.408
M1-/T1+/AA	17 (7.5)	4 (5.6)	0.509 (0.181–1.431)	0.201
M1-/T1+/AG+GG	34 (15.0)	9 (12.7)	0.499 (0.239–1.042)	0.064
M1+/T1-/AA	13 (5.7)	3 (4.2)	0.547 (0.166–1.801)	0.321
M1+/T1-/AG+GG	9 (4.0)	4 (5.6)	1.129 (0.387–3.295)	0.825
M1-/T1-/AA	3 (1.3)	0	—	—
M1-/T1-/AG+GG	26 (11.5)	5 (7.7)	0.317 (0.120–0.836)	**0.02**

Bold, significant association (*P*<0.05); CI, Confidence interval; HR, Hazard ratio; 1.0 (Reference); (+), Present; (^_^), Null;

^a^Adjusted for age, stage and histopathology.

## Discussion

The most important prognostic factors in cervical cancer patients primarily treated with chemo-radiation are stage and tumor histology [21]. However, the prognostic information provided by tumor stage and histology are not to an extent that they can be used to meet current requirements for optimal therapy. The toxicity and cost of additional therapy can only be justified in the subset of patients at high risk of local as well as distant relapse. Although chemoradiation improves survival and locoregional control, the 5 year overall survival rate being ~60% [22].

The effects of chemotherapy and radiotherapy are potentially modified by GST enzymes [23–24]. An attempt was made to identify genetic polymorphisms in *GST* gene which can be a predictive marker for response and survival to cisplatin based concomitant chemoradiation (CRT) in patients with advanced cervical cancer. Cisplatin (CDDP), commonly used in the treatment of cervical cancer is detoxified partly by *GST* enzyme.It was confirmed that increased GST expression in tumor cells determines resistance to cisplatin and other platinum-compounds [25–27]. The direct involvement of *GSTP1* in the detoxification of CDDP is by forming CDDP-GSH adducts [28–29].

Many studies suggested that GST plays an important role in development of tumor cell resistance to treatment. Reactive oxygen species (ROS) is generated during the treatment from both regimens *viz*. chemotherapy and radiotherapy. ROS is also generated as part of the cytotoxic activity of chemotherapeutic agents [30–31]. Radiotherapy may kill cancer cells either directly through effect on target molecules or indirectly through ROS. These ROS can damage cells, proteins and DNA or other cellular molecules.

Two possible mechanisms have been suggested for an association between GST genotypes and treatment outcome, one involving differences in carcinogen damage to DNA and the other, differences in detoxification by treatment agents or GST-mediated protection against oxidative damage during treatment [23]. In addition to GST, genetic polymorphisms in DNA repair genes are also known to be associated with cervical cancer and treatment outcomes of CRT [32–33]. One of the important DNA repair genes influencing response of cervical carcinoma to neoadjuvant chemotherapy was *XRCC1* gene variant at codon 399 [34]. Another gene variant *RAD51* G172T also showed association in overall survival of cervical cancer patients treated with CRT [2].

Many studies showed an association between polymorphisms of *GSTM1* and *T1* with treatment outcome in patients with breast cancer or childhood leukemia [7,35] but no association was found in colorectal cancer [11]. No individual effect of *GSTM1* or *T1* genotypes was found but in combination, null genotypes of both were associated with poor survival in ovarian cancer [36–37]. In the present study, a reduced hazard of death and better overall survival was observed among CRT treated women with null genotype of *GSTM1* (M1-*)*, particularly in combination with *GSTT1* null (M1-/T1-) and *GSTP1* (M1-/AG+GG) (Table 2). These results correspond with the hypothesis that the treatment would be more successful among patients with less or no GST activity.

According to Stanulla et al., the risk of recurrence was reduced among children with *GSTM1* null, *GSTT1* null and *GSTP1* GG genotypes in acute lymphoblastic leukemia [38]. This probably resulted in reduced or no enzymatic activity. The present study also showed a similar result *i*.*e*. a significant reduction in recurrence among women only with *GSTM1* null genotype (M1-).

An association of *GSTP1* with both acquired and intrinsic resistance to cisplatin was observed in human colon cancer cells [39]. Oesophageal cancer patients with decreased intratumoral*GSTP1* levels and those treated with FU/cisplatin based chemoradiotherapy showed better survival [40]. However, McLeod et al., did not find a correlation between *GSTP1*-A313G polymorphism and response to oxaliplatin based chemotherapy in colorectal cancer [41]. The present study demonstrated that *GSTP1* AG+GG polymorphism individually did not show any association with survival but in combination with *GSTM1* null genotype (M1-/AG+GG) and both *GSTM1* and *GSTT1* null genotype (M1-/T1-/AG+GG) showed reduced hazard ratio which means a better overall survival.

## Conclusion

Our results demonstrated that *GST* genotypes correlate with the clinical outcome of CRT treated cervical cancer patients. The interindividual difference results in differential activity of GST enzymes thus having impact on treatment outcome. However, the genetic variability in *GST* polymorphisms among individuals may be studied in larger patient populations in order to develop useful therapeutic regimens.
